# 

**Published:** 1896-02

**Authors:** 


### CONTENTS.

ORIGINAL ARTICLES—

Adenoids in the Naso-Pharynx. By Edward J. Bermingham, A. M., M. D., New York .......... 53
Chloroform in Labor. By Dr. Ben. H. Brodnax, Brodnax, La.. 59
A Rare Syphilitic Infection. By William S. Gottheil, M. D., New York .......... 64
Hæmorrhoids. By J. P. Stewart, M. D., Attalla, Ala .......... 65
New York Letter .......... 67

SOCIETY REPORTS—

Atlanta Society of Medicine .......... 70

SELECITONS AND ABSTRACTS—

A Case of Phthisis Apparently Cured. By William Peffer, M. D. .... 80
Consent in Surgical Operations .... 82
Suggestion in the Operation of Circumcision .... 85

BOOK REVIEWS .... 87
EDITORIAL .... 89
NOTES .... 92
CORRESPONDENCE .... 93
SPECIAL NOTES .... 96
Prescriptions .... 102

